# Supplementary material for: Design principles for electronic charge transport in solution-processed vertically stacked 2D perovskite quantum wells
Source: Nat Commun. 2018 May 30;9:2130. doi: 10.1038/s41467-018-04430-2 (PMC5976721; doi:10.1038/s41467-018-04430-2)
Supplement: Supplementary file 1 — Supplementary Information [file 41467_2018_4430_MOESM1_ESM.pdf]

Supplementary Information

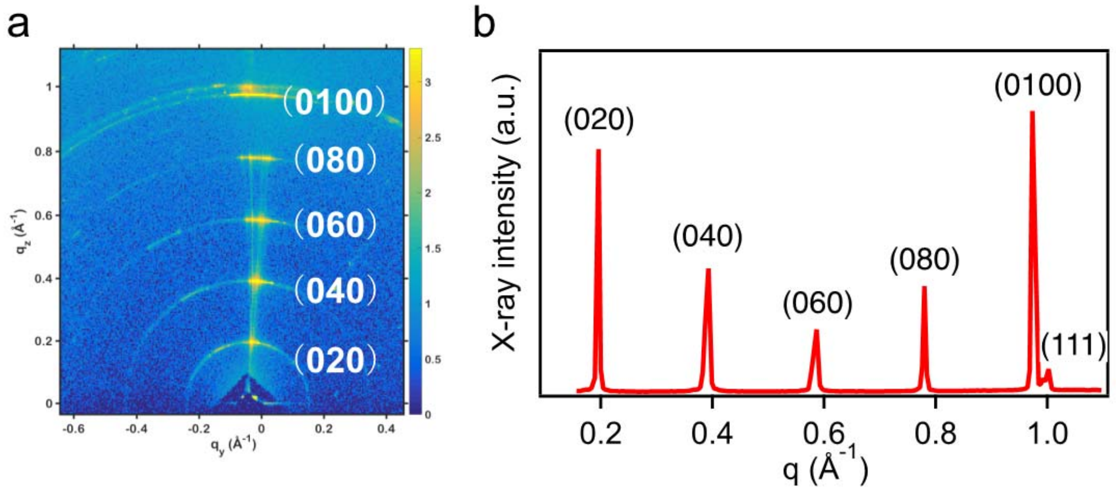

Supplementary Figure 1. X-ray diffraction for synthesized Pb_4 powder done under synchrotron source. **a**, GIWAXS map and **b**, line-cut for Pb_4 powder.

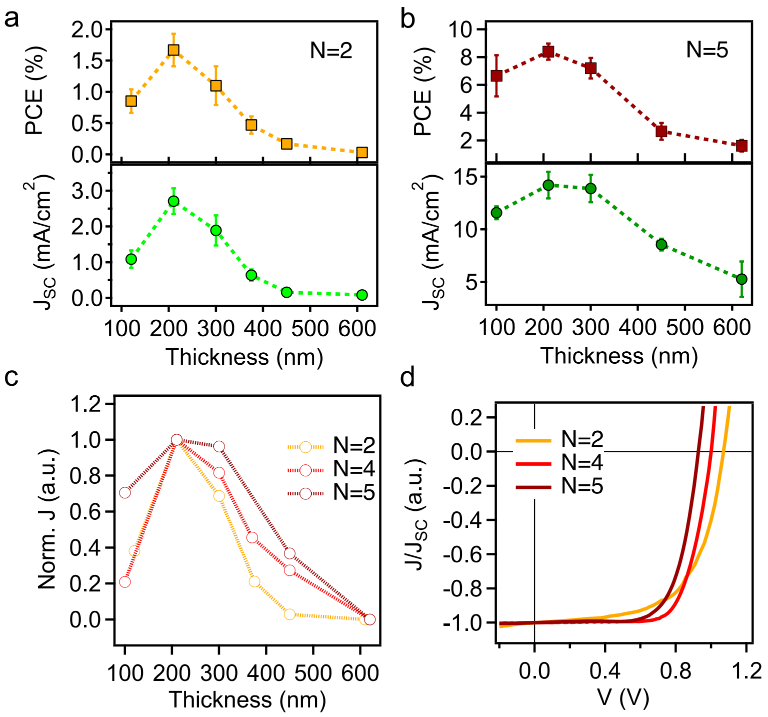

Supplementary Figure 2. Thickness dependent study for various n numbered layered perovskites. a-b, average power conversion efficiency (PCE) and short circuit current density (J_{SC}) as a function of absorbing layer thicknesses for n=2 and n=5 compounds, respectively. The samples were produced under the same processing condition. **c**, Normalized J_{SC} versus thickness plots for three different n-numbered layered perovskite devices. **d**, Normalized *J-V* characteristics for those devices with optimized thickness (220 nm).

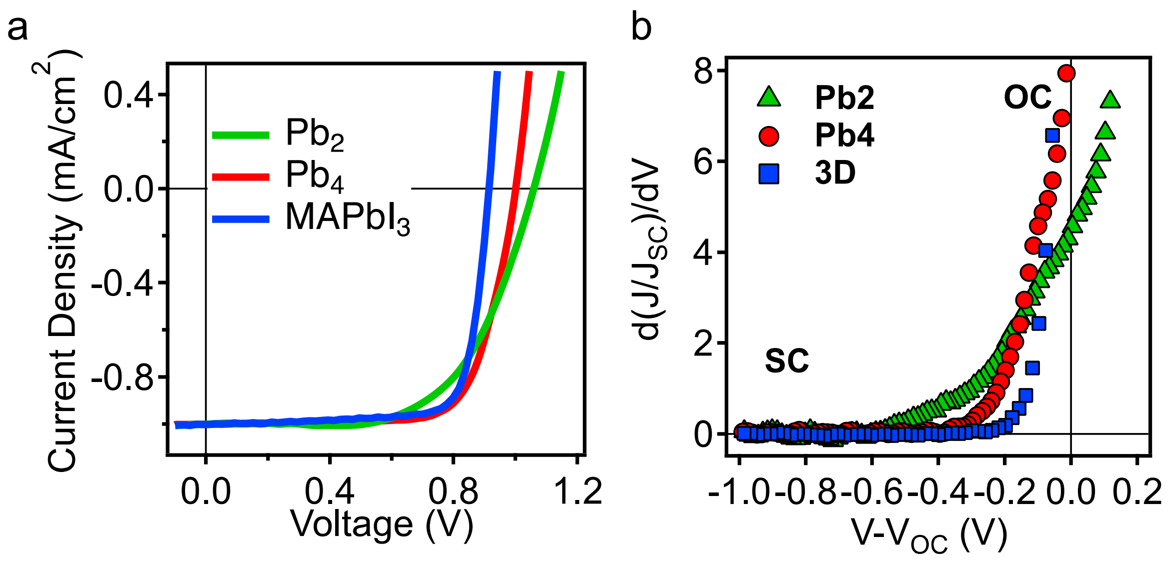

Supplementary Figure 3. *J-V* curve slopes for different n numbers. a, normalized JV curves under 1-Sun illumination for device fabricated with Pb_2 (green) and Pb_4 (red) as absorber fabricated with the same film thickness (220 nm); as a comparison, 3D device is also plotted in the same figure (blue). **b,** the slopes were examined by taking the first order of derivative from a and plotted as a function of field.

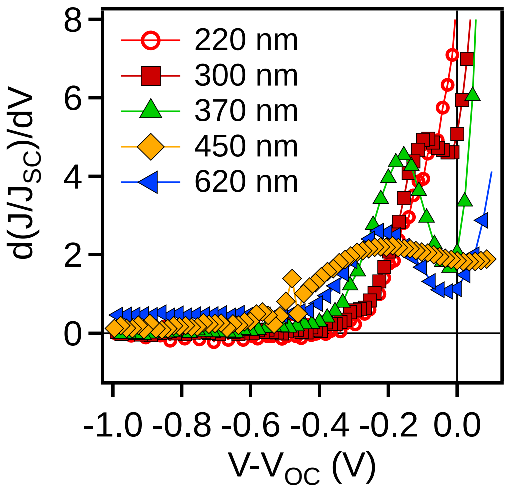

Supplementary Figure 4. Thickness dependent *J-V* curve slopes as a function of internal voltage obtained from Fig.2a. The JV slopes are extracted by taking the 1-st order derivative of the light *J-V* curves normalized by the J_{SC} value at each thickness. The derivatives are plotted as a function of internal voltage (V-V_{OC}) in MS Fig. 2.

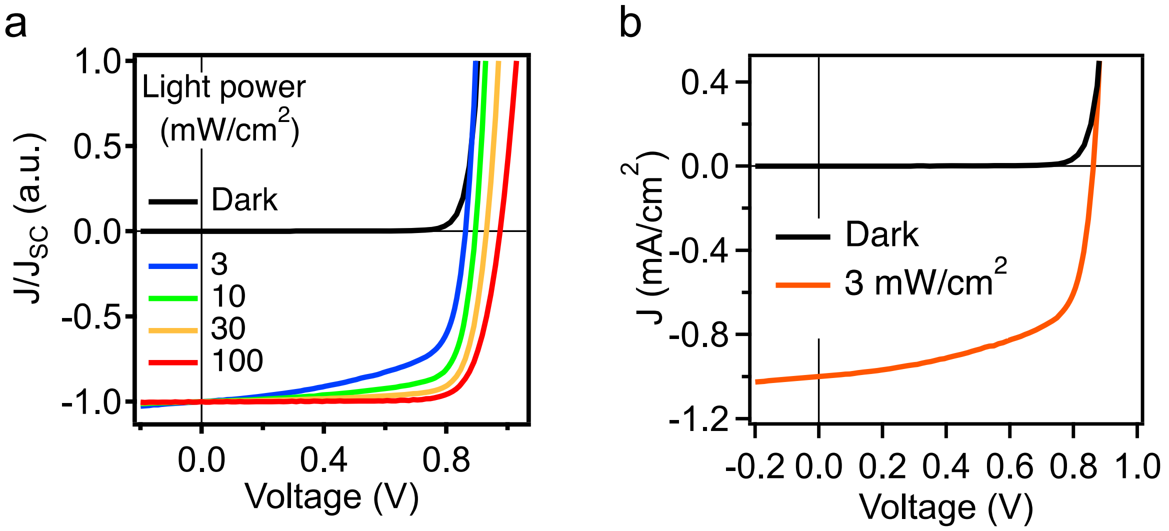

Supplementary Figure 5. *J-V* characteristics for device with 220 nm absorber thickness. a, dark and light *J-V* curves under various intensities, light curves were normalized by the short circuit current value at each intensity. **b**, dark and light J-V curves measured under lowest intensity used for this study for 220-nm device, where photocurrent is 2 orders of magnitude higher than the dark current, and dark injection near OC is not significant.

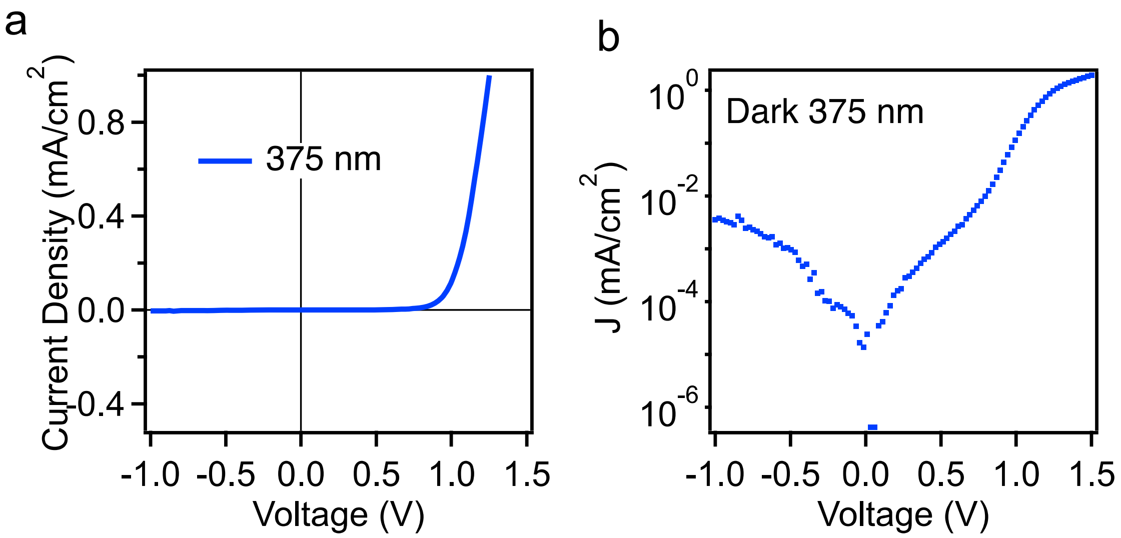

Supplementary Figure 6. Dark curve for 375 nm device in **a,** linear scale and **b,** logarithm scale. The dark curve for 375 nm device is 2-order of magnitude lower than the photocurrent of this device under the lowest illumination condition.

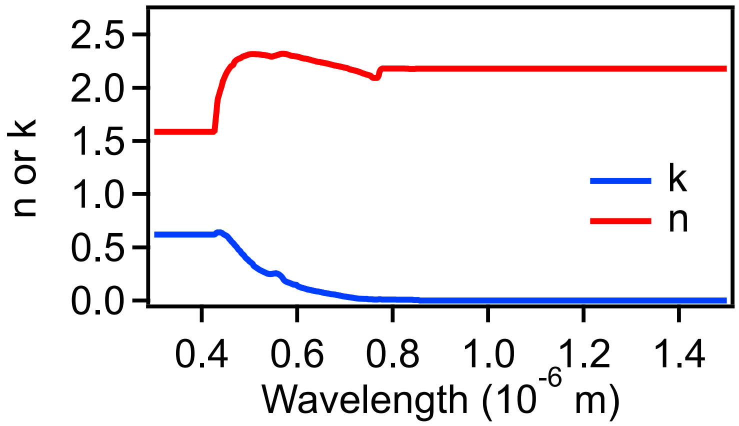

Supplementary Figure 7. Optical constant. (*n*, *k* values) as a function of wavelength for layered perovskite thin film used in modeling.

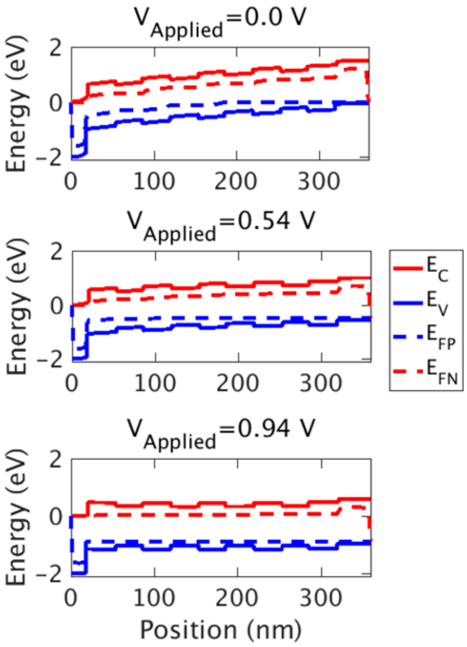

Supplementary Figure 8. The energy band diagram for 300 nm perovskite thickness at three different external bias conditions.

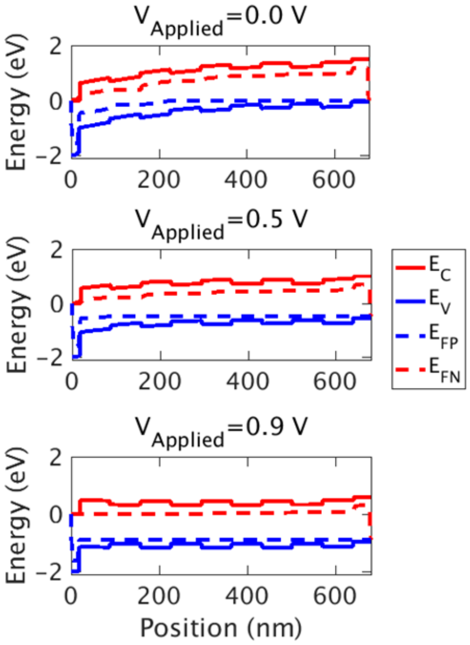

Supplementary Figure 9. The energy band diagrams for 620 nm perovskite thickness at three different external bias conditions.

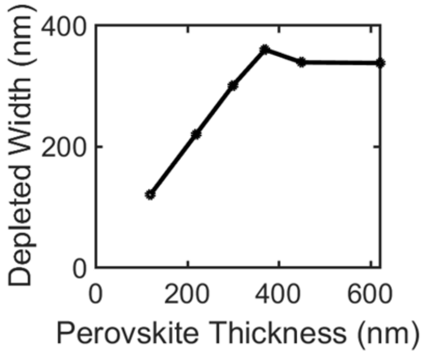

Supplementary Figure 10. Depletion width as a function of perovskite layer thickness. The device is fully depleted for up to 300 nm.

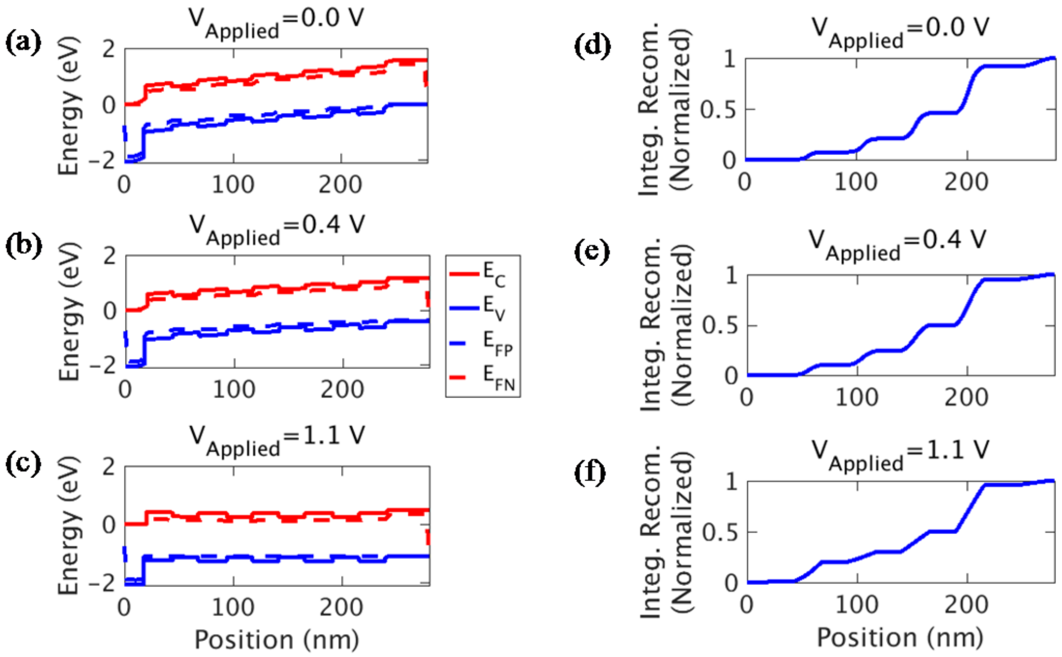

Supplementary Figure 11. Energy band diagram and normalized total recombination at T=150 K. At short circuit(**a,d**), maximum power point (**b,e**) and (**c,f**) open circuit. More than 95% of total recombination occurs inside the quantum wells. Low temperature does not provide enough thermal energy for carriers to leave the wells efficiently.

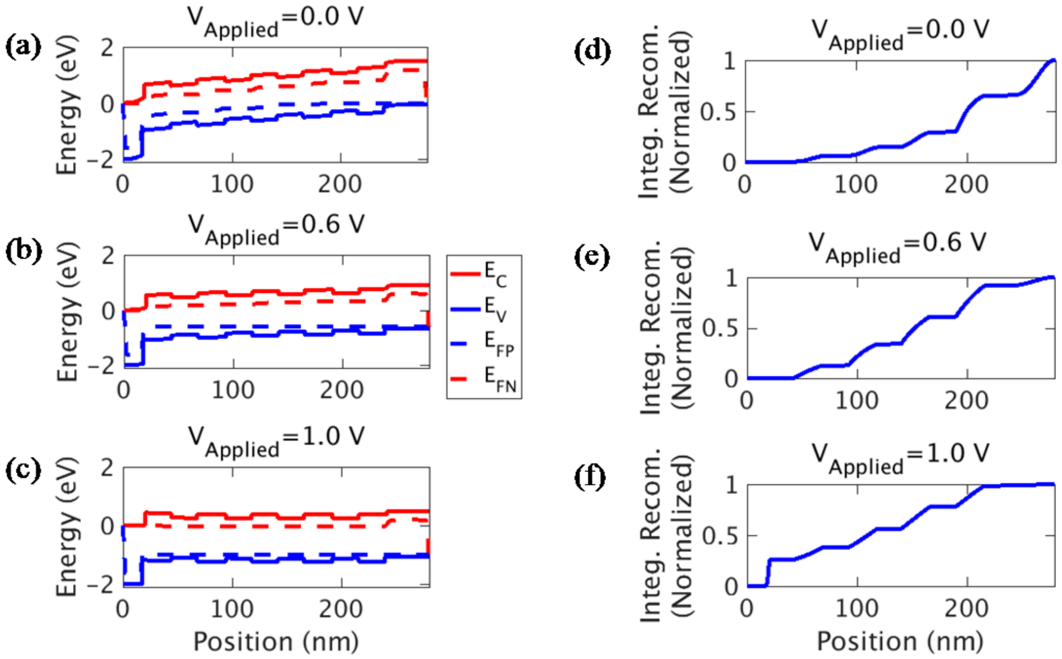

Supplementary Figure 12. Energy band diagram and normalized total recombination at T=300 K. At short circuit (**a,d**), maximum power point (**b,e**)and open circuit(**c,f**). Less than 75% of total recombination occurs inside the quantum wells since thermal energy helps the carriers to escape the wells.

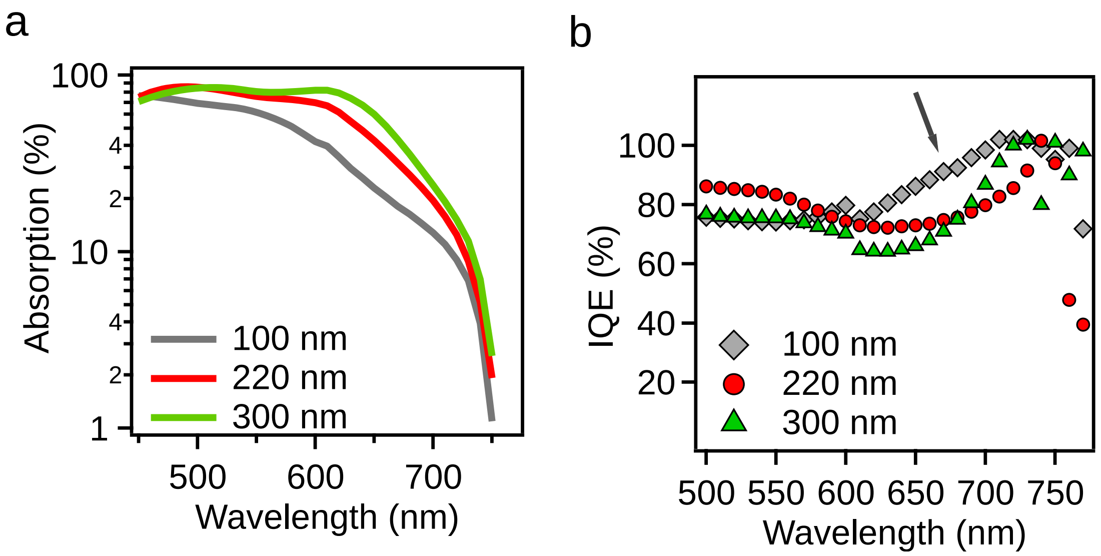

Supplementary Figure 13. Internal quantum efficiency. a, device absorption spectra measured in reflection mode. **b**, IQE spectra for those devices.

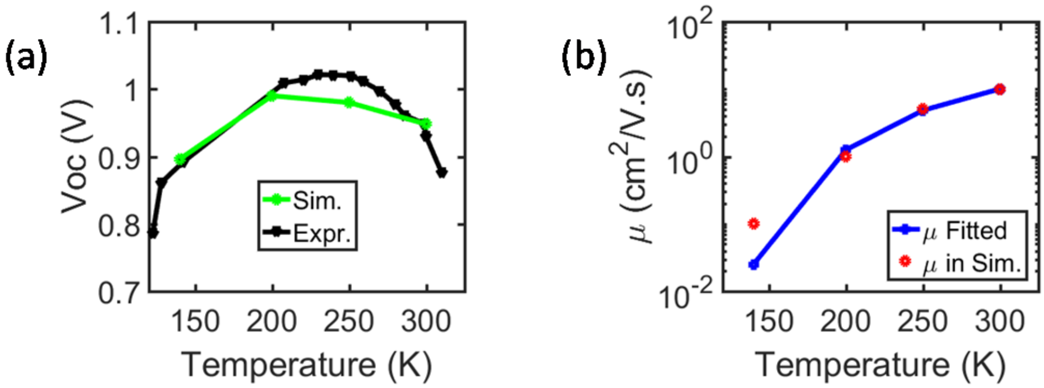

Supplementary Figure 14. a, Experimental and simulated V_{OC} as a function of temperature after considering the change in band gap and mobility at low temperature; **b**, The mobility values used in the drift-diffusion simulation were fitted by the Gaussian-disorder mobility model.

Supplementary Note 1. X-ray diffraction

The powder was synthesized according to literature published method[1], we took the powder for structural characterization under synchrotron source. The results are shown in Supplementary Figure 1. All the characteristic low angle peaks are observed in the compound as labeled as $(0k0)$ peak[2,3], which is a clear indication of pure n=4 phase formation.

Supplementary Note 2. Thickness dependence for other n-numbered RP perovskite cells

For other n-numbered layered perovskite devices, we expect several changes to the system: number of potential barriers (reduced size of conducting channel), band gap energy and field distribution profile. To investigate the impact of n-number on the device behavior, we conducted a thickness dependent study for two other n-numbered layered perovskite devices in the same device configuration described in the manuscript. We chose one compound with lower n value (n=2) and one higher n value (n=5) to compare with the n=4 device reported in the manuscript. The results are shown in Supplementary Figure 2.

From the results in Supplementary Figure 2, we found that the thickness dependence of the device performance for all the layered systems follow similar trend. The optimized thickness for all three compounds is roughly around 200 nm. This is reasonable given that the structures for those n=2, 4, 5 compounds are similar, the electrical transport properties are not expected to differ dramatically. Therefore, a field dependent carrier collection through multiple potential barriers across the film remains to be the major limitation for the solar cell performance. Further, from Supplementary Figure 2d, we observe a sharper decay in the case of lower n number as compared to higher n numbers. This is consistent with our proposed mechanism where lower n-

numbered thin film is more insulating likely due to a reduced conducting slab size. This also reflects in the normalized *J*-*V* characteristics in Supplementary Figure 2d, where a more pronounced field dependent slope from maximum power point towards short circuit can be observed in n=2 device, while the other two are more flattened.

Supplementary Note 3. Comparison between different systems

We compare three different systems using Pb_2, Pb_4 and 3D perovskite as absorber by taking the normalized light *J*-*V* curves in Supplementary Figure 3. The slopes of those *J*-*V* curves are extracted in Supplementary Figure 3b. It is clear that the most significant difference between the 2D and 3D system is the low field photocurrent collection, as demonstrated in the main manuscript. Furthermore, as n number reduces, such low field slope gets more flattened, indicating an increased number of barriers in the system.

Supplementary Note 4. Device simulation

Optical Modeling. Optical absorption in different layers of the PV cell is calculated by the full-wave solution of Maxwell's equations with the input of AM 1.5G illumination. The materials in different layers of the cell are characterized by their respective absorption coefficient and refractive indices. Those parameters are either obtained from literature [4,5] or measured data by the authors. Transfer matrix method (TMM) [6] calculations are used for the optical studies of the planar cell structure. In this approach, the entire solar cell stack, including the contact layers, is modeled using a series of interface and phase matrices. The central quantity which is calculated in this approach is the spatially-resolved optical absorbance [A(λ,r)] inside various layers of the cell. The wavelength range of 300–1500 nm has been used for our calculations (see

Supplementary Figure 7). The spatially resolved absorption profile is integrated over the wavelength range to create the generation profile for the electron and holes for self-consistent carrier transport simulation, as described below.

Self-consistent transport simulation. The transport of charged carriers (electrons and holes) is modeled by generalized drift-diffusion formalism[7]. Photo-generation is calculated from the optical absorption profile (integrated over the wavelengths) discussed previously. The electron and hole transport inside the cell is simulated by a self-consistent solution of Poisson and continuity equations by a commercial grade device simulator MEIDCI™ [8]. The generation term in the e–h continuity equations is calculated from the solution of photo-generated profile. The recombination term in the continuity equation consists of direct and Shockley-Read-Hall (SRH) recombinations with the respective lifetimes taken from the literature. In the calculation, we did not account for hot electron effects. The excitons – if any – are presumed to dissociate into free electron and hole pairs immediately after generation. For all of the simulations (including different thicknesses), we consider four quantum wells throughout the film thickness, which gives 4 lower band gap wells encased within 5 high band gap barriers. We assume that these wells have similar thickness and are randomly distributed within the absorber. The thickness of each potential well is thus the thickness of full film divided by nine. For example, for perovskite thickness of 620 nm, 450 nm, 370 nm, 300 nm, 220 nm, and 120 nm, the thickness of quantum well is 68.6 nm, 50 nm, 41.1 nm, 33.3 nm, 24.4 nm, and 13.3 nm respectively.

The model equations are summarized in Supplementary Table 1 and the simulation parameters are given in Supplementary Table 2. The parameters with references are taken from the

literature; the rest are assumed to match the data for 2D perovskite layer. To reproduce the trend of experimental results we used quantum wells with depth of 130 meV at both conduction and valence bands. Therefore, the bandgap in the quantum well is 260 meV lower than the barrier regions.

Electrical field profile for different film thicknesses. To examine the evolution of electrical field distribution with increasing film thickness, we have simulated the band diagrams based on the charge density profile from *J-V* curve fittings for three different film thicknesses: 200 nm, 300 nm (Supplementary Figure 8) and 620 nm (Supplementary Figure 9).

From the results in Supplementary Figure 8-9, we found the depletion region width for those layered perovskite cells in this device configuration used in current study as well as our previous published study (Reference 16 in MS) are in the range of 200~300 nm, where internal electrical field drops uniformly across two electrodes at short circuit condition.

Temperature Dependence of Recombination. Thermal energy of the carriers plays an important role in overcoming the barrier. Higher temperature helps the carriers to escape the quantum well easier. As shown in Supplementary Figure 11-12 for two temperatures of T=150 K and T=300 K the recombination becomes more prominent at the interface for higher temperature (see Supplementary Figure 12). The carriers can go over the quantum well barriers but not over the interfacial energy misalignment at Electron Transport Material (ETM)/perovskite interface at 20 nm at T=300 K. Therefore, less than 75% of total recombination occurs inside the quantum wells compared to more than 95% of recombination at T=150 K.

Supplementary Note 5. Electric filed distribution

To obtain the electrical field distribution, we consider a planar solar cell geometry studied in this work (Fig. 1a in the MS), where the light illustrates the transparent electrodes with PEDOT:PSS as hole transport layer (HTL). The electric field thus decreases monotonically from HTL to the PCBM (ETL) side (Supplementary Figure 10). The depleted width is defined as the location within the absorber where the internal field is reduced to 25% of the highest internal field at the short-circuit condition. For the cases of 220 nm and 300 nm, the perovskite layer is fully depleted based on our previous experimental results [9]. Supplementary Figure 10 shows a plot of the depletion width as a function of thickness of the perovskite layer. The slight peak at 380nm is related to numerical uncertainty of the simulation. The cut-off of 25% is arbitrary, other cut-off values would produce comparable results.

When the thickness continues to grow (e.g. 620 nm in Supplementary Figure 9), the field tends to be flattened in the middle region of the film, beyond the depletion region near the electron contact. These data are consistent with our thickness dependent *J-V* curve analysis in MS Figure 2. In MS Figure 2b, the slope near short circuit (SC) can be correlated to the charge collection efficiency under short circuit condition. This charge collection efficiency depends strongly on the drift field in the absorber layer. We find that the charge collection in the layered perovskite system begins to be field dependent above 350 nm indicated by the increased value in the *J-V* slope, suggesting the field strength is greatly reduced above that threshold and carriers undergo recombination before being collected.

Supplementary Note 6. Internal quantum efficiency

From the absorption spectra in Supplementary Figure 13a, we found the internal absorption increases as film thickness grows from 100 nm to 300 nm as expected. This is benefited from thicker film that absorbs more photon at the band edge as well as optical density change when thickness changes. This suggests that the absorption is indeed incomplete for film thinner than 200 nm. In Supplementary Figure 13b, we found in all three cases (100 nm, 220 nm and 300 nm thick), the IQE values are above 80% and the band edge at near IR regime are much stronger than that near the visible range. This is likely due to the photo-excited carrier nature in the layered perovskites where highly bonded carriers are formed near high energy with binding energy greater than 100 meV [12,16]. Those bonded carriers generated at this energy may not be collected efficiently before recombination. The near IR excitation forms weakly bonded carriers that can be collected efficiently [12] leading to much higher IQE values. However, we emphasize that the IQE for 100nm thin film device near 500~770 nm range (arrow in Supplementary Figure 13b) are indeed higher than thicker device. This suggests that the collections are much more efficient in the thinner device, where internal field are stronger.

Supplementary Note 7. Temperature dependent Open circuit voltage

The increase in V_{OC} at high temperature range follows the predicted trend, but the saturation near low temperature range was not reproduced by simulation. To understand such discrepancy, we have therefore considered two more effects: a) mobility and b) band gap change with temperature.

Band gap change with temperature. The open circuit voltage for a planar solar cell should increase with reduced temperature in classical semiconducting systems, where the trap assisted recombination and electron-phonon interactions are greatly suppressed[10]. Hybrid perovskites are unique semiconductors where the band gap energy in fact reduces at low temperature, this is true for 3D perovskites[11] as well as 2D perovskites[12]. Based on our previous study, the optical band gap energy systematically reduces with temperature that will significantly affect the open circuit voltage at low temperature.

Carrier mobility. In a system with disordered energetic landscapes, it was proposed in organic photovoltaic[13] system that the carrier mobility follows a Gaussian-disorder mobility model[14] of the form

$$\mu = \mu_\infty \exp\left(-\left(c\frac{\sigma}{kT}\right)^2\right)$$

Here, μ_∞ is asymptotic high-temperature mobility limit, σ Gaussian-disorder width, and previous work in related material has found c~2/3[15].

Based on those two factors, we have thus refined our model in the new set of simulations, and results are summarized in Supplementary Figure 14. In the refinement, we took the temperature dependent band gap change from our previous work[12]. And the Gaussian-disorder mobility has been incorporated for transport within the barrier and the quantum well regions. With $\sigma = 50\,meV$, we find $\mu_\infty = 53.4, c = 0.668$ (Supplementary Figure 14b). In Supplementary Figure 14a shows the simulated V_{OC} (T) curve, that reproduces the saturation near low temperature range.

Supplementary Table 1. Equations for Carrier Transport

Poisson Equation:

$$\epsilon_r \epsilon_0 \nabla^2 \psi = -q\ (n_h - n_e)$$

Continuity:

$$\nabla J_{e,h} = \left(G_{e,h} - R_{e,h}(n_e, n_h)\right)$$

Drift-Diffusion:

$$J_{e,h} = \mu_{e,h} n_{e,h} (-\nabla \psi) \pm D_{e,h} \nabla n_{e,h}$$

Recombination:

$$R_{e,h}(n_e, n_h) = B(n_e n_h - n_i^2) + \frac{n_e n_h - n_i^2}{\tau(n_e + n_h)}$$

Supplementary Table 2. Absorber Parameters (Perovskite) [17-19]

Symbol	Description	Parameter value
$L_{Absorber}$	Thickness of absorber layer	220 nm
μ_e	Electron mobility in absorber	10.0 (cm^2/V.s)
μ_h	Hole mobility in absorber	10.0 (cm^2/V.s)
τ_e	Electron lifetime in absorber	10 (ns)
τ_h	Hole lifetime in absorber	10 (ns)
$LUMO$	Lowest Unoccupied Molecular Orbital	3.73 (eV)
E_G	Band gap of absorber	1.62 (eV)
ε_r	Relative dielectric constant	25
N_A	Self-Doping concentration (P-type)	1.0 e16 (#/cm^3)

Supplementary Table 3. Electron Transport Material Parameters (ETM = PCBM) [20-23]

Symbol	Description	Parameter value
L_{ETM}	Thickness of ETM layer	20 nm
μ_e	Electron mobility in ETM	1e-2 (cm^2/V.s)
μ_h	Hole mobility in ETM	1e-2 (cm^2/V.s)
τ_e	Electron lifetime in ETM	1000 (ns)
τ_h	Hole lifetime in ETM	1000 (ns)
$LUMO$	Lowest Unoccupied Molecular Orbital	4.17 (eV)
E_G	Band gap of ETM	2.0 (eV)
ε_r	Relative dielectric constant	4
N_D	Self-Doping concentration (N-type)	5.0 e17 (#/cm^3)

Supplementary Table 4. Hole Transport Material Parameters (HTM = PEDOT:PSS) [24,25]

Symbol	Description	Parameter value
L_{HTM}	Thickness of HTM layer	40 nm
μ_e	Electron mobility in HTM	9e-3 (cm^2/V.s)
μ_h	Hole mobility in HTM	9e-3 (cm^2/V.s)
τ_e	Electron lifetime in HTM	1000 (ns)
τ_h	Hole lifetime in HTM	1000 (ns)
$HUMO$	Highest Occupied Molecular Orbital	5.18 (eV)
E_G	Band gap of HTM	1.55 (eV)
ε_r	Relative dielectric constant	3
N_A	Self-Doping concentration (P-type)	3.0 e17 (#/cm^3)

Supplementary References

1. Cao, D. H., Stoumpos, C. C., Farha, O. K., Hupp, J. T. & Kanatzidis, M. G. 2D Homologous Perovskites as Light-Absorbing Materials for Solar Cell Applications. *J. Am. Chem. Soc.* **137**, 7843-7850 (2015).
2. Stoumpos, C. C. *et al.* High Members of the 2D Ruddlesden-Popper Halide Perovskites: Synthesis, Optical Properties, and Solar Cells of (CH3(CH2)3NH3)2(CH3NH3)4Pb5I16. *Chem* **2**, 427-440 (2017).
3. Stoumpos, C. C. *et al.* Ruddlesden–Popper Hybrid Lead Iodide Perovskite 2D Homologous Semiconductors. *Chem. Mater.* **28**, 2852-2867 (2016).
4. Gevaerts, V. S., Koster, L. J. A., Wienk, M. M. & Janssen, R. A. J. Discriminating between Bilayer and Bulk Heterojunction Polymer:Fullerene Solar Cells Using the External Quantum Efficiency. *ACS Appl. Mater. Interfaces* **3**, 3252-3255 (2011).
5. Hoppe, H., Sariciftci, N. S. & Meissner, D. Optical constants of conjugated polymer/fullerene based bulk-heterojunction organic solar cells. *Mol. Cryst. Liq. Cryst.* **385**, 113-119 (2002).
6. Pettersson, L. A. A., Roman, L. S. & Inganäs, O. Modeling photocurrent action spectra of photovoltaic devices based on organic thin films. *J. Appl. Phys.* **86**, 487-496 (1999).
7. Pierret, R. *Advanced Semiconductor Fundamentals*. (Prentice Hall, 2002).
8. (Synopsys, 2010).
9. Tsai, H. *et al.* High-efficiency two-dimensional Ruddlesden–Popper perovskite solar cells. *Nature* **536**, 312-316 (2016).

10. Löper, P. *et al.* Analysis of the Temperature Dependence of the Open-Circuit Voltage. *Energy Procedia* **27**, 135-142 (2012).
11. Dar, M. I. *et al.* Origin of unusual bandgap shift and dual emission in organic-inorganic lead halide perovskites. *Sci. Adv.* **2**, e1601156 (2016).
12. Blancon, J.-C. *et al.* Extremely efficient internal exciton dissociation through edge states in layered 2D perovskites. *Science* **355**, 1288-1292 (2017).
13. Chavali, R. V. K. *et al.* A Generalized Theory Explains the Anomalous Suns– $V_{{rm{oc}}}$ Response of Si Heterojunction Solar Cells. *IEEE J. Photovolt.* **7**, 169-176 (2017).
14. Yang, W., Luo, Y., Guo, P., Sun, H. & Yao, Y. Leakage Current Induced by Energetic Disorder in Organic Bulk Heterojunction Solar Cells: Comprehending the Ultrahigh Loss of Open-Circuit Voltage at Low Temperatures. *Phys. Rev. Appl.* **7**, 044017 (2017).
15. Bässler, H. Charge Transport in Disordered Organic Photoconductors a Monte Carlo Simulation Study. *physica status solidi (b)* **175**, 15-56 (1993).
16. Blancon, J.-C. *et al.* Unusual thickness dependence of exciton characteristics in 2D perovskite quantum wells. *arXiv:1710.07653* (2017).
17. Stranks, S. D. *et al.* Electron-Hole Diffusion Lengths Exceeding 1 Micrometer in an Organometal Trihalide Perovskite Absorber. *Science* **342**, 341-344 (2013).
18. Brivio, F., Walker, A. B. & Walsh, A. Structural and electronic properties of hybrid perovskites for high-efficiency thin-film photovoltaics from first-principles. *APL Mater.* **1**, 042111 (2013).
19. Schulz, P. *et al.* Interface energetics in organo-metal halide perovskite-based photovoltaic cells. *Energy Environ. Sci.* **7**, 1377-1381 (2014).
20. Trukhanov, V. A., Bruevich, V. V. & Paraschuk, D. Y. Effect of doping on performance of organic solar cells. *Phys. Rev. B* **84**, 205318 (2011).
21. Larson, B. W. *et al.* Electron Affinity of Phenyl–C61–Butyric Acid Methyl Ester (PCBM). *J. Phys. Chem. C* **117**, 14958-14964 (2013).
22. Guan, Z.-L. *et al.* Direct determination of the electronic structure of the poly(3-hexylthiophene):phenyl-[6,6]-C61 butyric acid methyl ester blend. *Org. Electron.* **11**, 1779-1785 (2010).
23. Veldman, D. *et al.* Compositional and Electric Field Dependence of the Dissociation of Charge Transfer Excitons in Alternating Polyfluorene Copolymer/Fullerene Blends. *J. Am. Chem. Soc.* **130**, 7721-7735 (2008).
24. Lenz, A., Kariis, H., Pohl, A., Persson, P. & Ojamäe, L. The electronic structure and reflectivity of PEDOT:PSS from density functional theory. *Chem. Phys.* **384**, 44-51 (2011).
25. Rutledge, S. A. & Helmy, A. S. Carrier mobility enhancement in poly(3,4-ethylenedioxythiophene)-poly(styrenesulfonate) having undergone rapid thermal annealing. *J. Appl. Phys.* **114**, 133708 (2013).
